# Processing of DNA Topoisomerase II–DNA–Protein Crosslinks Associated With Anticancer Drugs

**DOI:** 10.1111/gtc.70076

**Published:** 2025-12-17

**Authors:** Ryo Sakasai, Kuniyoshi Iwabuchi

**Affiliations:** ^1^ Department of Biochemistry I Kanazawa Medical University Kahoku Japan

**Keywords:** DPC, DSB, NHEJ, proteasome, TOP2, VCP

## Abstract

During cell division and gene expression, the DNA double‐helical structure unwinds, thereby generating torsional stress. DNA topoisomerases are enzymes that relieve this stress. During this process, topoisomerases form temporary covalent bonds with the phosphate backbone of DNA, generating DNA strand breaks and relieving torsional stress. Topoisomerases then dissociate from DNA after rejoining the DNA breaks. Torsional stress associated with replication or transcription is primarily relieved by topoisomerase I (TOP1) and II (TOP2). Some anticancer drugs targeting topoisomerases, known as topoisomerase poisons, trap the topoisomerase reaction intermediates and cause DNA strand breaks bearing topoisomerase–DNA–protein crosslinks (TOP–DPCs). TOP1 poisons, such as camptothecin, cause DNA single‐strand breaks bearing TOP1–DPCs, which are converted to DNA double‐strand breaks (DSBs) when they collide with DNA replication forks. In contrast, TOP2 poisons, such as etoposide, directly induce DSBs in TOP2–DPCs. However, to elicit a DSB response, TOP2–DPC must first be removed from the DSB ends. Cells possess various pathways to remove TOP2–DPC, and these pathways are thought to function in coordination depending on the situation. This review summarizes these sophisticated TOP2–DPC removal pathways and discusses the clinical applications of TOP2 poison as an anticancer drug, as well as the related challenges.

## Graphical abstract

Topoisomerase II (TOP2) relieving DNA torsional stress is trapped by TOP2 poisons, such as etoposide, inducing DNA double‐strand breaks (DSBs) bearing TOP2–DNA–protein crosslinks (TOP2–DPCs), which must be removed before DSB repair. This review summarizes the TOP2–DPC resolution processes mediated by multiple pathways, including proteolysis and nucleolysis.

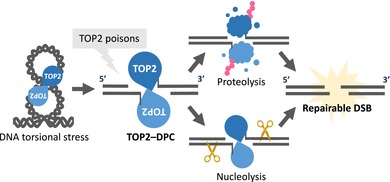

## 1 Introduction

The cells in our bodies contain DNA, which carries genetic information and is stably maintained inside their nuclei. Depending on the state of the cell, DNA undergoes significant changes in its topology. When cells divide to proliferate, they must replicate the DNA to double its quantity. Additionally, the transcription of many genes is spatially and temporally regulated to maintain cellular functions. DNA replication and transcription require the unwinding of double‐stranded DNA. After replication or transcription, the DNA must return to a double‐stranded state. Changes in the topology of the DNA double helix may influence chromatin structure and potentially affect gene expression and genomic stability. Alterations in the topology of the DNA double helix occur naturally during the unwinding and renaturation processes, and DNA topoisomerases relieve such torsional stresses. Vertebrates, including humans, have six DNA topoisomerase genes: *TOP1*, *TOP2A*, *TOP2B*, *TOP3A*, *TOP3B*, and *TOP1MT* (seven if the meiosis‐specific *SPO11* is included). TOP1MT functions in the mitochondria, whereas the others function in the nucleus. TOP1, TOP3α, and TOP3β are classified as type I topoisomerases, whereas TOP2α and TOP2β are classified as type II topoisomerases. All topoisomerases transiently cleave DNA by forming covalent bonds between a tyrosine residue of the topoisomerase and the phosphate backbone of the DNA (Pommier et al. 2022). After the topological stress is relieved, the DNA strand breaks are rejoined and the topoisomerase dissociates from the DNA.

There are inhibitors that target TOP1 and TOP2, known as TOP1 and TOP2 poisons, respectively. These inhibitors trap reaction intermediates and induce DNA strand breaks bearing topoisomerase–DNA–protein crosslinks (TOP–DPCs). TOP–DPCs have been widely referred to as TOP–DNA cleavage complexes (TOPccs). In this review, TOPccs trapped by TOP poisons are denoted as TOP–DPCs to distinguish them from spontaneous TOPccs that occur during the native reaction process. TOP2 poisoning induces DSBs bearing TOP2–DPCs. Although the DSB repair and TOP2–DPC removal are closely related, they are distinct processes, each mediated by particular factors, and must be considered separately. Recent studies have revealed that efficient DSB repair requires TOP2–DPC resolution, which is regulated in a complex manner by multiple pathways. Therefore, in this review, we particularly focus on TOP2 and summarize how cells respond to TOP2–DPC induced by TOP2 poisons such as etoposide.

## 2 DNA Topoisomerase II

**FIGURE 1:**
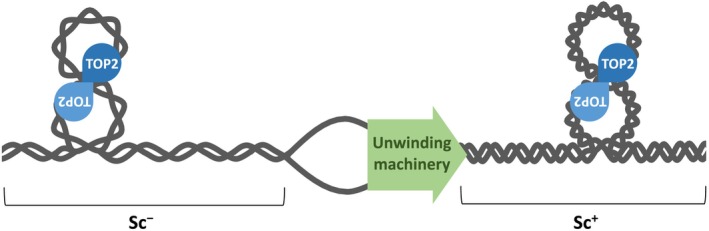
TOP2 and DNA supercoils associated with DNA transactions. During DNA replication and transcription, DNA unwinding inevitably alters DNA topology, generating DNA supercoils. The DNA double helix becomes overwound ahead of the unwinding machinery, generating positive supercoils (Sc^+^), whereas it becomes underwound behind the unwinding machinery, generating negative supercoils (Sc^−^). These DNA topological alterations are resolved by TOP2.

The unwinding of double‐helical DNA during replication and transcription elicits alterations in DNA topology. When the double helix unwinds, the genomic DNA cannot rotate freely, resulting in a situation referred to as positive supercoils (Sc^+^), where the pitch of the twisting becomes denser ahead of the unwinding machinery. Contrastingly, behind the unwinding machinery, the DNA twists loosen, leading to a state referred to as negative supercoils (Sc^−^) (Figure 1). TOP2 can resolve some types of DNA topological stresses, including Sc^+^ and Sc^−^, and as well as tangles involving two strands of DNA, such as DNA catenanes and knots (Nitiss 2009a; Pommier et al. 2022). TOP2 uses adenosine triphosphate (ATP) hydrolysis as an energy source to catalyze the cleavage and rejoining of double‐stranded DNA. TOP2 temporarily forms a phospho‐tyrosyl bond with the 5′ end of the DNA when cleavage occurs. TOP2 functions as a homodimer; therefore, a DNA DSB is induced by the two monomers, each cleaving one strand of DNA. After a DSB is generated, the torsional stress is relieved by the passage of another DNA strand through the DSB, and the DSB is quickly rejoined.

In vertebrates, including humans, two paralogs of the TOP2 gene exist: *TOP2A* and *TOP2B*, which encode the TOP2α and TOP2β proteins, respectively. Although TOP2α and TOP2β share similar catalytic properties, they have different functions (Nitiss 2009a). TOP2β is expressed throughout the cell cycle, whereas TOP2α levels peak during the S and G2/M phases of the cell cycle and are associated with cell proliferation (Heck et al. 1988; Kimura et al. 1994). In addition to its role in resolving torsional stress associated with replication fork progression, TOP2α plays a significant role in chromosomal condensation and separation during mitosis (McClendon et al. 2005; Li et al. 2008; Farr et al. 2014; Nielsen et al. 2020). TOP2β activity is primarily associated with transcription. It participates in the regulation of chromatin structure around promoter regions of specific genes and transcription elongation (Ju et al. 2006; Lyu et al. 2006; King et al. 2013; Madabhushi et al. 2015; Uuskula‐Reimand and Wilson 2022; Yao et al. 2025). In addition, it has also been reported that TOP2α is involved in transcriptional regulation by interacting with RNA polymerase II (Mondal and Parvin 2001; Herrero‐Ruiz et al. 2021). Furthermore, TOP2α has been reported to be necessary for the expression of specific genes in embryonic stem cells and during their differentiation (Thakurela et al. 2013). These findings indicate that TOP2α also contributes to the regulation of transcriptional activity in concert with TOP2β. In addition to TOP2, TOP1 also contributes to the resolution of the supercoils associated with replication or transcription. The coordinated actions of TOP2α, TOP2β, and TOP1 efficiently resolve the torsional stress generated by DNA transactions.

## 3 Targeting TOP2 as Anticancer Drugs

Drugs that target TOP2 are widely used in chemotherapy to treat blood cancers, such as leukemia and lymphoma, as well as solid tumors, such as breast and lung cancers. These drugs induce DNA damage by compromising TOP2 function and cause cell death in cancer cells. These include TOP2 poisons and catalytic inhibitors that interfere with TOP2 activity via mechanisms different from those of the poisons (Nitiss 2009b; Pommier et al. 2010; Vann et al. 2021; Jang et al. 2025).

TOP2 poisons such as etoposide and teniposide trap TOP2 reaction intermediates and accumulate TOP2–DPCs bound to the 5′ end of DSBs. Additionally, the DNA intercalator‐type TOP2 poisons, doxorubicin (an anthracycline) and mitoxantrone (an anthraquinone), also cause the accumulation of TOP2–DPCs (Nitiss 2009b). However, these poisons are associated with severe side effects such as cardiotoxicity and secondary cancers accompanied by chromosomal translocations (Cowell and Austin 2012; Zhang et al. 2012; Pendleton et al. 2014; Linders et al. 2024). Conversely, TOP2 catalytic inhibitors reduce enzyme activity. ICRF‐193 and ICRF‐187 inhibit TOP2's ATP hydrolysis, allowing TOP2 to bind to DNA and inhibit the catalytic cycle without forming TOP2ccs. ICRF‐187 is clinically used in combination with doxorubicin and other anthracycline‐based agents to reduce cardiotoxicity (Ishida et al. 1995; Larsen et al. 2003; Lyu et al. 2007; Szponar et al. 2024).

Cells attempt to repair DSBs induced by TOP2 poisons. The repair of TOP2 poison–induced DSBs mainly relies on nonhomologous end‐joining (NHEJ), one of the major pathways for DSB repair, and defects in NHEJ factors, such as DNA ligase IV and DNA‐dependent protein kinase (DNA‐PK), greatly increase cellular sensitivity to etoposide (Adachi et al. 2003; Willmore et al. 2004; Maede et al. 2014). However, the trapped TOP2 by TOP2 poisons such as etoposide becomes an obstacle to DNA repair mechanisms. An in vitro study has shown that DNA‐PK fails to activate in response to DSBs bound by TOP2 (Martensson et al. 2003). Therefore, quickly removing the trapped TOP2 proteins and exposing the free 5′ ends of DNA is necessary for efficient repair. Meanwhile, ectopic rejoining of DSBs can cause leukemia‐inducing chromosomal translocations, which are significant side effects of TOP2 poisons (Cowell and Austin 2012; Olmedo‐Pelayo et al. 2020). A deeper understanding of how TOP2–DPCs are processed could help reduce these side effects and improve the efficacy of chemotherapy involving TOP2 poisons.

## 4 Proteolytic Resolution of TOP2–DPC

**FIGURE 2:**
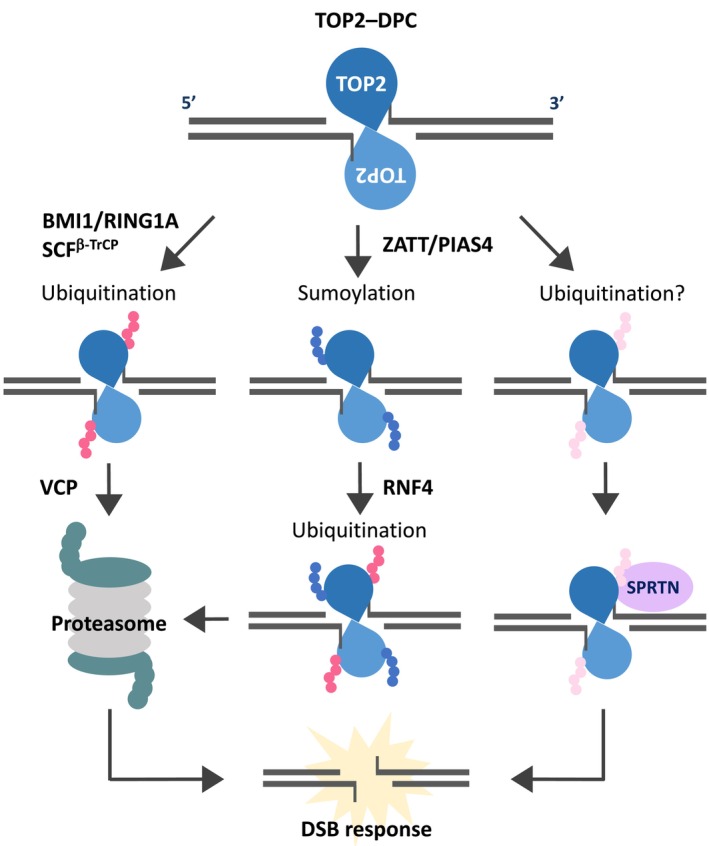
Pathways for trapped TOP2 proteolysis. TOP2–DPCs caused by TOP2 poisons undergo ubiquitination by BMI1/RING1A or SCF^β‐TrCP^, leading to proteasomal degradation and generation of a naked DSB that can trigger DSB responses (left pathway). Alternatively, ZATT and PIAS4 mediate SUMOylation of the trapped TOP2, promoting RNF4‐dependent ubiquitination and subsequent proteasomal proteolysis (central pathway). Additionally, SPRTN contributes to the removal of TOP2–DPCs via a proteasome‐independent mechanism, which may be enhanced by TOP2 ubiquitination (right pathway).

The resolution of TOP2–DPCs induced by TOP2 poisons can be divided into two stages. The first stage is the degradation of TOP2, an obstacle bound to the 5′ end of the DSB. The second stage involves the processing of the DSB ends for subsequent repair. Each of these stages is controlled by multiple pathways (Figure 2).

Exposure to TOP2 poisons leads to the formation of TOP2–DPCs, which triggers TOP2 degradation. Previous studies have shown that proteasome inhibition delays the removal of TOP2–DPCs by TOP2 poisons, leading to their accumulation. This suggests that TOP2 poisons‐induced degradation of TOP2 is proteasome‐dependent (Mao et al. 2001; Xiao et al. 2003; Zhang et al. 2006; Azarova et al. 2007). However, proteasome inhibition reduces the appearance of etoposide‐induced γH2AX, a phosphorylated form of the histone variant H2AX, which is a well‐known DSB marker (Rogakou et al. 1998; Paull et al. 2000; Mao et al. 2001; Zhang et al. 2006; Sciascia et al. 2020; Swan et al. 2020). Additionally, proteasome inhibition suppresses the activation of ataxia–telangiectasia mutated (ATM) and ATM‐ and Rad3‐related (ATR) proteins, which function as DNA damage sensors and key regulators of the DNA damage response (DDR), along with their downstream signaling pathways (Zhang et al. 2006; Robison et al. 2007; Fan et al. 2008). These findings suggest that proteasome activity is necessary for converting “low‐reactive DSBs” concealed by trapped TOP2 into “high‐reactive naked DSBs” capable of inducing DSB responses.

Etoposide has been reported to cause the accumulation of phosphorylated forms of RNA polymerase II including Ser2 phosphorylation on its C‐terminal domain (Ban et al. 2013), which results from the arrest of transcription elongation. This suggests that TOP2–DPCs induce transcriptional stress through collisions between TOP2–DPCs and the transcription machinery. Proteasomal processing of TOP2–DPCs is thought to be transcription‐dependent; the collision of the transcription machinery with TOP2–DPCs may act as a trigger to induce TOP2 degradation (Mao et al. 2001; Xiao et al. 2003; Zhang et al. 2006; Fan et al. 2008; Tammaro et al. 2013). Indeed, genome‐wide analyses have revealed that TOP2–DPC resolution is accelerated at transcriptionally active loci (Canela et al. 2019). By contrast, DNA replication has not been shown to affect TOP2 degradation (Fan et al. 2008), suggesting that TOP2–DPC resolution is not associated with DNA replication. However, as DNA replication affects etoposide sensitivity and the processing of DSBs, it may influence chemotherapy sensitivity through a mechanism distinct from transcription‐dependent TOP2–DPC resolution (Fan et al. 2008; Tammaro et al. 2013).

Valosin‐containing protein (VCP) (also known as p97) is a molecular chaperone of the proteasome with AAA+ ATPase activity. VCP utilizes the energy from ATP hydrolysis to induce conformational changes in ubiquitinated substrate proteins, thereby contributing to protein degradation and the regulation of protein–protein interactions (Meyer et al. 2012). VCP has also been reported to play a role in the DDR by detaching specific DNA repair proteins, including Ku70/80 and DNA‐PK, from chromatin (Acs et al. 2011; Bergink et al. 2013; Jiang et al. 2013; van den Boom et al. 2016). In budding yeast cells with compromised Cdc48 (the yeast homolog of VCP/p97), accumulation of ubiquitinated TOP2 has been reported (Wei et al. 2017), suggesting that TOP2 degradation is regulated by VCP. Similar to the effects of proteasome inhibition, inhibition of VCP activity results in the delayed removal of TOP2–DPC after etoposide treatment and reduces ATM and H2AX phosphorylation in human cells (Swan et al. 2021; Sakasai et al. 2025). In addition, DNA‐PK activation is strongly inhibited by proteasome or VCP inhibition. Consistent with this, DSB repair efficiency after etoposide treatment is also suppressed by VCP inhibition (Sakasai et al. 2025). As DNA‐PK is activated by binding to DNA ends in a Ku protein‐dependent manner (Dvir et al. 1992; Gottlieb and Jackson 1993), DNA‐PK is considered to be significantly affected by trapped TOP2 at the DSB ends. The reduction in DSB repair efficiency may result from a combination of physical obstructions in DNA‐PK end‐binding and rejoining reactions by trapped TOP2. Furthermore, the conformational changes in trapped TOP2 by VCP are thought to play an important role not only in TOP2 degradation but also in the cleanup step for a protein adduct–free DSB ends, as discussed in Section 5.

For the degradation of trapped TOP2 via the proteasome and VCP, the trapped TOP2 must undergo ubiquitination. BMI1 and RING1A, which form a ubiquitin ligase for the polycomb repressive complex 1 (PRC1), are reported to be involved in TOP2α degradation induced by TOP2 poisons (Alchanati et al. 2009; Swan et al. 2020). BMI1 and RING1A directly ubiquitinate TOP2α. Additionally, the SCF (SKP1–Cul1–F‐box) complex containing β‐TrCP has also been reported to ubiquitinate trapped TOP2β. After ATM activation in response to the TOP2 poison teniposide, casein kinase 1‐mediated TOP2β phosphorylation leads to SCF^β‐TrCP^‐mediated TOP2β ubiquitination and degradation (Shu et al. 2020). Furthermore, ZATT (ZNF451) and PIAS4, both SUMO ligases, SUMOylate TOP2. The SUMOylated TOP2 is then ubiquitinated by the sumoylation‐dependent ubiquitin ligase RNF4, which promotes its degradation by the proteasome (Schellenberg et al. 2017; Sun et al. 2020) (Figure 2).

In addition, ubiquitination factors not involved in TOP2–DPC resolution have also been identified. APC/C‐Cdh1 controls the stability of TOP2α by ubiquitinating TOP2α (Eguren et al. 2014). Additionally, BRCA1 and RNF168 are known to regulate decatenation activity by adding a ubiquitin chain to TOP2α via lysine 63, which is different from the lysine 48‐linked ubiquitin chains related to protein degradation (Guturi et al. 2016).

SPRTN is a DNA‐binding metalloprotease that is reported to be involved in resolving DPCs induced by formaldehyde or the TOP1 poison camptothecin (Lopez‐Mosqueda et al. 2016). SPRTN is also thought to promote the removal of DPCs at replication forks by its recruitment to DPCs through the interactions with proteins in the replication machinery (Vaz et al. 2016; Morocz et al. 2017; Larsen et al. 2019). SPRTN deficiency sensitizes cells to etoposide and increases the level of etoposide‐induced TOP2–DPCs. Additionally, SPRTN binds to TOP2 and degrades it in the presence of ubiquitin in vitro (Lopez‐Mosqueda et al. 2016). SPRTN is known to be ubiquitinated, and its ubiquitination state regulates chromatin binding (Stingele et al. 2016). The ubiquitination of target proteins is reported to enhance SPRTN‐mediated proteolysis (Durauer et al. 2025). These findings imply that SPRTN‐mediated TOP2–DPC resolution is regulated by the ubiquitination state of both SPRTN and TOP2 (Figure 2). However, the relationship between SPRTN and replication during TOP2–DPC resolution is not well understood. RNF4 is also involved in SPRTN‐mediated DPC degradation via DPC SUMOylation. In RNF4‐deficient cells, etoposide‐induced SPRTN activation depends on DNA replication (Weickert et al. 2023). This suggests that, under certain conditions, TOP2–DPC degradation by SPRTN may be coupled with DNA replication.

## 5 DSB End Cleanup for DSB Repair

**FIGURE 3:**
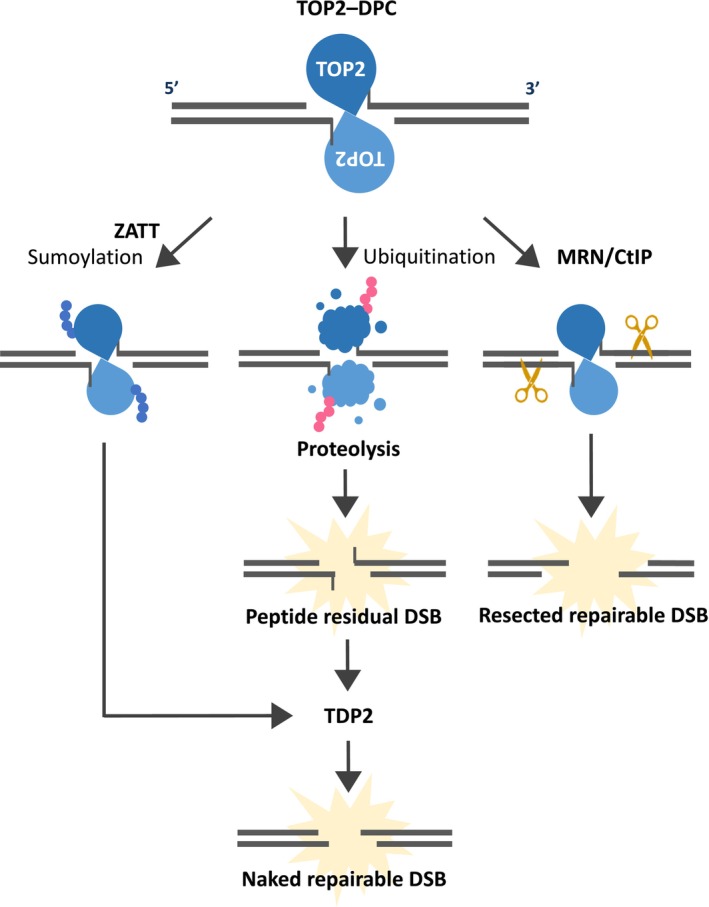
Pathways for end cleanup of DSBs bearing TOP2. TOP2–DPCs undergo ubiquitination followed by proteasomal degradation; however, the peptide containing the TOP2 tyrosine residue remains at the DSB end. This residual peptide is removed by TDP2, making the DSB end repairable (central pathway). ZATT‐dependent SUMOylation can also facilitate end processing by TDP2, independent of proteasomal degradation of TOP2 (left pathway). Alternatively, the DSB end bearing TOP2 is resected by MRN (MRE11–RAD50–NBS1)/CtIP nucleases, generating a repairable DSB through excision of the TOP2–DPC (right pathway).

After TOP2 trapping and subsequent degradation by the proteasome or SPRTN, a peptide containing the TOP2 tyrosine residue remains at the DSB end. This degradation increases the accessibility of Tyrosyl DNA phosphodiesterase 2 (TDP2) to the DSB ends (Gao et al. 2014; Lee et al. 2018), and TDP2 cleaves the covalent bond between this peptide and DNA (Cortes Ledesma et al. 2009; Zeng et al. 2011). Through the reaction by TDP2, the DSB bearing TOP2–DPC is converted into a free DSB end that can be joined by NHEJ (Schellenberg et al. 2016) (Figure 3). As TDP2 deficiency induces etoposide sensitivity, removing the peptide from the DSB end is thought to be crucial for DSB repair (Zeng et al. 2011; Gomez‐Herreros et al. 2013). However, TDP2 deficiency does not significantly impact the sensing or signaling of DSBs in response to etoposide (Sakasai et al. 2025), suggesting that as long as TOP2 is degraded, DDR can be activated regardless of the presence of the residual peptide. Recently, Saha et al. reported that SPRTN targets TOP3α–DPC and has an epistatic relationship with TDP2 (Saha et al. 2023), suggesting that TDP2 removes the remaining peptides after SPRTN degrades TOP3α. In TOP2–DPC, TDP2 may work in coordination with SPRTN through a similar mechanism to TOP3α–DPC resolution (Figure 2).

The cleanup of DSB ends by TDP2 is promoted by TOP2 degradation. However, recent reports have shown that ZATT promotes the direct removal of TOP2–DPC by TDP2 through a proteasome‐independent mechanism (Schellenberg et al. 2017). SUMOylation of trapped TOP2 by ZATT is thought to induce conformational changes in TOP2, enabling TDP2 to access the binding site of TOP2 and facilitate cleavage of TOP2–DPC (Figure 3). Recently, RAD54L2 was identified as a factor that binds to SUMOylated TOP2. D'Alessandro et al. and Zhang et al. showed that RAD54L2, using its ATPase activity, is involved in a ZATT‐dependent, proteasome‐independent TOP2–DPC removal pathway (D'Alessandro et al. 2023; Zhang et al. 2023). A novel RAD54L2‐mediated pathway has been proposed to dissociate TOP2 from chromatin in a TDP2‐independent manner.

Another pathway for DSB end cleanup is the nucleolytic pathway. In this pathway, TOP2 proteins are removed by nucleases such as CtIP and MRE11 (Figure 3). These nucleases are involved in DNA‐end resection during DSB repair (Sartori et al. 2007; Nicolette et al. 2010; Garcia et al. 2011). DNA‐end resection is an early stage of DSB repair via homologous recombination (HR). However, DNA‐end resection has been reported to be involved in the repair of TOP2 poison–induced DSB via NHEJ in the G1 and G0 phases, in which HR does not function (Quennet et al. 2011; Akagawa et al. 2020). Increases in TOP2 poison–induced TOP2–DPCs and cytotoxicity have been reported in cells depleted of MRE11 and/or CtIP (Nakamura et al. 2010; Lee et al. 2012; Aparicio et al. 2016; Hoa et al. 2016). In addition, MRE11 dysfunction has been reported to cause TOP2–DPC accumulation, even in the absence of TOP2 poisons, suggesting the importance of MRE11 in removing naturally generated TOP2ccs (Hoa et al. 2016). MRE11 is a well‐known nuclease that functions as a heterotrimer with NBS1 and RAD50 during DNA‐end resection (Paull 2018). Similar to that in MRE11‐depleted cells, TOP2–DPC accumulation was observed in NBS1‐depleted cells, suggesting that the MRE11–RAD50–NBS1 (MRN) complex functions in the nucleolytic removal pathway of TOP2‐DPC (Sun et al. 2022). MRE11‐mediated removal of TOP2–DPC is BRCA1‐dependent, and the interaction between BRCA1 and CtIP has been shown to be important for this pathway (Nakamura et al. 2010; Aparicio et al. 2016; Sasanuma et al. 2018). In the repair of DSB induced by TOP2 poisons, MRE11 and TDP2 function via separate pathways, suggesting that the nucleolytic and proteolytic pathways additively promote NHEJ (Hoa et al. 2016). By contrast, VCP has been suggested to play an important role in both pathways. Overexpression of MRE11 can remove TOP2–DPCs induced by etoposide, even in the presence of proteasome inhibitors, but not in the presence of VCP inhibitors (Sun et al. 2022). Thus, the VCP‐mediated conformational change in TOP2 is considered necessary for both the proteolytic pathway mediated by the proteasome/SPRTN and the nucleolytic pathway mediated by the MRN complex/CtIP.

## 6 Top2‐Targeting Chemotherapy and Carcinogenesis

Ectopic rejoining of DSBs causes chromosomal abnormalities such as chromosome translocation. This is a well‐known side effect of TOP2 poisons and is associated with secondary leukemia (Felix 1998; Mistry et al. 2005; Pendleton et al. 2014). For example, approximately 2%–9% of patients treated with etoposide develop acute myeloid leukemia (t‐AML) (Winick et al. 1993), which is associated with translocations involving the *MLL* (*KMT2A*) gene located at 11q23 and multiple fusion partner genes, such as *ENL*, *AF4*, *AF6*, and *AF9* (Felix 1998; Cowell et al. 2012). Additionally, many cases of primary infant leukemia exhibit the same chromosomal translocations as those observed in patients with TOP2 poison–induced t‐AML (Pendleton et al. 2014). The infant leukemia may also be caused by a similar mechanism mediated by spontaneous TOP2ccs.

Chromatin fibers form loop structures which are considered fundamental structural features for transcriptional regulation. At the base of the loops, distal chromosomal regions become proximate, increasing the proximity of genes to the distal chromosomal regions within the loop. Furthermore, multiple genes in close proximity are cooperatively transcribed. Such transcriptionally active sites, concentrated in specific nuclear regions, have been proposed as a transcription factory model. The concept of transcriptional concentrates, in which transcription domains aggregate via liquid–liquid phase separation, has also been accepted (Jha et al. 2022; Qi et al. 2025). Cowell et al. suggested that *MLL* and fusion partner genes are transcribed by a common transcription machinery, which may increase the proximity between *MLL* and fusion partner genes during transcription (Cowell et al. 2012). The proximity of genomic structures mediated by transcription underlies chromosomal translocations, and Meaburn et al. observed that gene fusions are more likely to occur when spatial proximity increases (Meaburn et al. 2007). Similar to the chromosomal rearrangements observed in leukemia, DSBs mediated by TOP2β are associated with the translocations of androgen‐responsive genes in prostate cancer. Genes such as *TMPRSS2* and *ERG*, which are well‐known fusion genes in prostate cancer, are thought to be transcribed by a common transcription apparatus that exhibits high spatial proximity. This situation creates an environment in which they are prone to chromosomal translocations (Kumar‐Sinha et al. 2008; Mani et al. 2009; Haffner et al. 2010). Spatial proximity depends on chromosome structure, such as transcription‐associated chromosome loops, which are formed in a cohesin‐ and CCCTC‐binding factor (CTCF)‐dependent manner. TOP2β is also known to colocalize with cohesin and CTCF in the loop structure (Uuskula‐Reimand et al. 2016; Canela et al. 2017). In fact, DSBs are frequently induced by etoposide in these regions (Canela et al. 2017; Gothe et al. 2019). As treatment with TOP2 poisons can induce DSBs when distal genes are adjacent, it can increase the risk of chromosomal rearrangements (Canela et al. 2019; Gothe et al. 2019).

Proteasome inhibitors, such as bortezomib, are clinically used as anticancer agents for multiple myeloma, mantle cell lymphoma, and other conditions (Manasanch and Orlowski 2017; Sin and Man 2021). VCP inhibitors are also currently being studied in clinical trials as potential anticancer agents (Kilgas and Ramadan 2023). Because proteasome and VCP inhibition suppress DDR and DSB repair caused by TOP2 poisons, it might reasonably be concluded that TOP2 poisons could enhance antitumor effects by cotreatment with proteasome or VCP inhibitors. Indeed, the combination of proteasome inhibitors with TOP2 poisons has been clinically evaluated for use in chemotherapy (Cowell and Austin 2012; Manasanch and Orlowski 2017; Dittus et al. 2018). Previous studies have shown that this combination enhances cell death (Ceruti et al. 2006; von Metzler et al. 2009; Aras and Yerlikaya 2016). However, recent studies at the cultured cell level have shown seemingly contradictory results, in which the combination of proteasome inhibitors and etoposide restores the survival of cells treated with etoposide alone (Lee et al. 2016; Sciascia et al. 2020; Sakasai et al. 2025). Sciascia et al. have shown that the timing of proteasome inhibition significantly affects the cytotoxicity of TOP2 poisons (Sciascia et al. 2020). They reported that TOP2 degradation is suppressed under preinhibited proteasome conditions, and that TOP2 enzymatic activity recovers after TOP2 poison removal, allowing the rejoining of DSBs and TOP2 dissociation from chromosomes. Therefore, although TOP2–DPC may accumulate temporarily in the presence of proteasome inhibitors, the undegraded TOP2 is reactivated and dissociates from the chromosome after TOP2 poison removal, thereby potentially leading to a reduction in the total amount of DSBs and cytotoxicity. Therefore, the development of effective combination therapies using proteasome inhibitors requires further investigation. Future studies into the toxicity of TOP2 poison–induced DSBs themselves, as well as their impact on chromosomal rearrangements as a side effect, may lead to new chemotherapy strategies.

## 7 Future Perspectives

The removal pathway of TOP2–DPCs caused by TOP2 poisons is controlled by several complementary pathways. This is crucial for maintaining genomic stability and serves as an effective defense mechanism against naturally occurring TOP2ccs. However, the complex relationship between these pathways makes it challenging to understand the effects of TOP2 poisons in cancer treatment. The existence of multiple repair pathways also provides a reliable defense mechanism for cancer cells. Clarifying the factors that affect pathway selection could lead to the development of combination therapies targeting these factors in conjunction with TOP2 poisons. However, the risk of secondary cancers caused by chromosomal rearrangements resulting from TOP2 poisoning remains a major issue. Further elucidation of the intracellular phenomena associated with TOP2–DPCs and verification of the appropriate balance between TOP2–DPC removal and DSB repair are necessary to improve chemotherapy using TOP2 poisons.

TOP1 poisons, such as irinotecan and topotecan, which are camptothecin derivatives, have also been used in chemotherapy. TOP1 poisons inhibit DNA replication and transcription by inducing TOP1–DPCs, and replication‐coupled DSBs are the cause of the cytotoxicity of TOP1 poisons (Venkatachalam and Kaufmann 2025). Similar to TOP2 poisons, proteasome inhibition suppresses the DSB response to TOP1 poisons (Sakasai et al. 2010). Distinct from TOP2 poisons, TOP1 poisons generate a single‐strand break (SSB) bearing TOP1 at its 3′ end. Therefore, although the SSB is converted to a DSB by collision with the replication fork, this DSB theoretically does not possess TOP1 at the end. However, in actual cellular conditions, the DSB end can somehow be covered by undegraded TOP1–DPC in the presence of a proteasome inhibitor. Various studies have been conducted on the combination of TOP1 poisons with proteasome inhibitors (Cusack et al. 2001; Ryan et al. 2006; Tang et al. 2014). Recently, research on TOP3 has progressed rapidly, and a TOP3β poison has also been identified (Wang et al. 2023). TOP3β is a type I topoisomerase and the only topoisomerase that acts on RNA. The newly identified TOP3β poison traps the reaction intermediates of TOP3β, primarily on RNA. Future research will likely clarify its biological responses and the specificity of its inhibitory properties. Therefore, it has potential as an anticancer or antiviral agent.

Topoisomerases are biologically intriguing enzymes with highly attractive activity directly involved in nucleic acid metabolism, which is a core cellular process. Therefore, topoisomerases still retain considerable potential as targets for anticancer and antiviral agents, and further research is required to expand their clinical application.

## Conflicts of Interest

The authors declare no conflicts of interest.

## Data Availability

Data sharing not applicable to this article as no datasets were generated or analysed during the current study.

## References

[gtc70076-bib-0001] Acs, K. , M. S. Luijsterburg , L. Ackermann , F. A. Salomons , T. Hoppe , and N. P. Dantuma . 2011. “The AAA‐ATPase VCP/p97 Promotes 53BP1 Recruitment by Removing L3MBTL1 From DNA Double‐Strand Breaks.” Nature Structural & Molecular Biology 18: 1345–1350. 10.1038/nsmb.2188 22120668

[gtc70076-bib-0002] Adachi, N. , H. Suzuki , S. Iiizumi , and H. Koyama . 2003. “Hypersensitivity of Nonhomologous DNA End‐Joining Mutants to VP‐16 and ICRF‐193: Implications for the Repair of Topoisomerase II‐Mediated DNA Damage.” Journal of Biological Chemistry 278: 35897–35902. 12842886 10.1074/jbc.M306500200

[gtc70076-bib-0003] Akagawa, R. , H. T. Trinh , L. K. Saha , et al. 2020. “UBC13‐Mediated Ubiquitin Signaling Promotes Removal of Blocking Adducts From DNA Double‐Strand Breaks.” IScience 23: 101027. 32283528 10.1016/j.isci.2020.101027 PMC7155233

[gtc70076-bib-0004] Alchanati, I. , C. Teicher , G. Cohen , et al. 2009. “The E3 Ubiquitin‐Ligase Bmi1/Ring1A Controls the Proteasomal Degradation of Top2alpha Cleavage Complex—A Potentially New Drug Target.” PLoS One 4: e8104. 19956605 10.1371/journal.pone.0008104 PMC2779455

[gtc70076-bib-0005] Aparicio, T. , R. Baer , M. Gottesman , and J. Gautier . 2016. “MRN, CtIP, and BRCA1 Mediate Repair of Topoisomerase II‐DNA Adducts.” Journal of Cell Biology 212: 399–408. 26880199 10.1083/jcb.201504005 PMC4754713

[gtc70076-bib-0006] Aras, B. , and A. Yerlikaya . 2016. “Bortezomib and Etoposide Combinations Exert Synergistic Effects on the Human Prostate Cancer Cell Line PC‐3.” Oncology Letters 11: 3179–3184. 27123085 10.3892/ol.2016.4340 PMC4841005

[gtc70076-bib-0007] Azarova, A. M. , Y. L. Lyu , C. P. Lin , et al. 2007. “Roles of DNA Topoisomerase II Isozymes in Chemotherapy and Secondary Malignancies.” Proceedings of the National Academy of Sciences of the United States of America 104: 11014–11019. 17578914 10.1073/pnas.0704002104 PMC1904155

[gtc70076-bib-0008] Ban, Y. , C. W. Ho , R. K. Lin , Y. L. Lyu , and L. F. Liu . 2013. “Activation of a Novel Ubiquitin‐Independent Proteasome Pathway When RNA Polymerase II Encounters a Protein Roadblock.” Molecular and Cellular Biology 33: 4008–4016. 23938298 10.1128/MCB.00403-13 PMC3811683

[gtc70076-bib-0009] Bergink, S. , T. Ammon , M. Kern , L. Schermelleh , H. Leonhardt , and S. Jentsch . 2013. “Role of Cdc48/p97 as a SUMO‐Targeted Segregase Curbing Rad51‐Rad52 Interaction.” Nature Cell Biology 15: 526–532. 23624404 10.1038/ncb2729

[gtc70076-bib-0010] Canela, A. , Y. Maman , S. N. Huang , et al. 2019. “Topoisomerase II‐Induced Chromosome Breakage and Translocation Is Determined by Chromosome Architecture and Transcriptional Activity.” Molecular Cell 75: 252–266.e8. 31202577 10.1016/j.molcel.2019.04.030 PMC8170508

[gtc70076-bib-0011] Canela, A. , Y. Maman , S. Jung , et al. 2017. “Genome Organization Drives Chromosome Fragility.” Cell 170: 507–521.e18. 28735753 10.1016/j.cell.2017.06.034 PMC6133249

[gtc70076-bib-0012] Ceruti, S. , A. Mazzola , and M. P. Abbracchio . 2006. “Proteasome Inhibitors Potentiate Etoposide‐Induced Cell Death in Human Astrocytoma Cells Bearing a Mutated p53 Isoform.” Journal of Pharmacology and Experimental Therapeutics 319: 1424–1434. 16971507 10.1124/jpet.106.109397

[gtc70076-bib-0013] Cortes Ledesma, F. , S. F. El Khamisy , M. C. Zuma , K. Osborn , and K. W. Caldecott . 2009. “A Human 5′‐Tyrosyl DNA Phosphodiesterase That Repairs Topoisomerase‐Mediated DNA Damage.” Nature 461: 674–678. 19794497 10.1038/nature08444

[gtc70076-bib-0014] Cowell, I. G. , and C. A. Austin . 2012. “Mechanism of Generation of Therapy Related Leukemia in Response to Anti‐Topoisomerase II Agents.” International Journal of Environmental Research and Public Health 9: 2075–2091. 22829791 10.3390/ijerph9062075 PMC3397365

[gtc70076-bib-0015] Cowell, I. G. , Z. Sondka , K. Smith , et al. 2012. “Model for MLL Translocations in Therapy‐Related Leukemia Involving Topoisomerase IIbeta‐Mediated DNA Strand Breaks and Gene Proximity.” Proceedings of the National Academy of Sciences of the United States of America 109: 8989–8994. 22615413 10.1073/pnas.1204406109 PMC3384169

[gtc70076-bib-0016] Cusack, J. C., Jr. , R. Liu , M. Houston , et al. 2001. “Enhanced Chemosensitivity to CPT‐11 With Proteasome Inhibitor PS‐341: Implications for Systemic Nuclear Factor‐kappaB Inhibition.” Cancer Research 61: 3535–3540. 11325813

[gtc70076-bib-0017] D'Alessandro, G. , D. A. Morales‐Juarez , S. L. Richards , et al. 2023. “RAD54L2 Counters TOP2‐DNA Adducts to Promote Genome Stability.” Science Advances 9: eadl2108. 38055822 10.1126/sciadv.adl2108 PMC10699776

[gtc70076-bib-0018] Dittus, C. , N. Grover , S. Ellsworth , X. Tan , and S. I. Park . 2018. “Bortezomib in Combination With Dose‐Adjusted EPOCH (Etoposide, Prednisone, Vincristine, Cyclophosphamide, and Doxorubicin) Induces Long‐Term Survival in Patients With Plasmablastic Lymphoma: A Retrospective Analysis.” Leukemia & Lymphoma 59: 2121–2127. 29303024 10.1080/10428194.2017.1416365

[gtc70076-bib-0019] Durauer, S. , H. S. Kang , C. Wiebeler , et al. 2025. “Allosteric Activation of the SPRTN Protease by Ubiquitin Maintains Genome Stability.” Nature Communications 16: 5422. 10.1038/s41467-025-61224-z PMC12279946 40691134

[gtc70076-bib-0020] Dvir, A. , S. R. Peterson , M. W. Knuth , H. Lu , and W. S. Dynan . 1992. “Ku Autoantigen Is the Regulatory Component of a Template‐Associated Protein Kinase That Phosphorylates RNA Polymerase II.” Proceedings of the National Academy of Sciences of the United States of America 89: 11920–11924. 1465419 10.1073/pnas.89.24.11920 PMC50669

[gtc70076-bib-0021] Eguren, M. , M. Alvarez‐Fernandez , F. Garcia , et al. 2014. “A Synthetic Lethal Interaction Between APC/C and Topoisomerase Poisons Uncovered by Proteomic Screens.” Cell Reports 6: 670–683. 24508461 10.1016/j.celrep.2014.01.017

[gtc70076-bib-0022] Fan, J. R. , A. L. Peng , H. C. Chen , S. C. Lo , T. H. Huang , and T. K. Li . 2008. “Cellular Processing Pathways Contribute to the Activation of Etoposide‐Induced DNA Damage Responses.” DNA Repair (Amst) 7: 452–463. 18206427 10.1016/j.dnarep.2007.12.002

[gtc70076-bib-0023] Farr, C. J. , M. Antoniou‐Kourounioti , M. L. Mimmack , A. Volkov , and A. C. Porter . 2014. “The Alpha Isoform of Topoisomerase II Is Required for Hypercompaction of Mitotic Chromosomes in Human Cells.” Nucleic Acids Research 42: 4414–4426. 24476913 10.1093/nar/gku076 PMC3985649

[gtc70076-bib-0024] Felix, C. A. 1998. “Secondary Leukemias Induced by Topoisomerase‐Targeted Drugs.” Biochimica et Biophysica Acta 1400: 233–255. 9748598 10.1016/s0167-4781(98)00139-0

[gtc70076-bib-0025] Gao, R. , M. J. Schellenberg , S. Y. Huang , et al. 2014. “Proteolytic Degradation of Topoisomerase II (Top2) Enables the Processing of Top2.DNA and Top2.RNA Covalent Complexes by Tyrosyl‐DNA‐Phosphodiesterase 2 (TDP2).” Journal of Biological Chemistry 289: 17960–17969. 24808172 10.1074/jbc.M114.565374 PMC4140274

[gtc70076-bib-0026] Garcia, V. , S. E. Phelps , S. Gray , and M. J. Neale . 2011. “Bidirectional Resection of DNA Double‐Strand Breaks by Mre11 and Exo1.” Nature 479: 241–244. 22002605 10.1038/nature10515 PMC3214165

[gtc70076-bib-0027] Gomez‐Herreros, F. , R. Romero‐Granados , Z. Zeng , et al. 2013. “TDP2‐Dependent Non‐Homologous End‐Joining Protects Against Topoisomerase II‐Induced DNA Breaks and Genome Instability in Cells and in Vivo.” PLoS Genetics 9: e1003226. 23505375 10.1371/journal.pgen.1003226 PMC3592926

[gtc70076-bib-0028] Gothe, H. J. , B. A. M. Bouwman , E. G. Gusmao , et al. 2019. “Spatial Chromosome Folding and Active Transcription Drive DNA Fragility and Formation of Oncogenic MLL Translocations.” Molecular Cell 75: 267–283.e12. 31202576 10.1016/j.molcel.2019.05.015

[gtc70076-bib-0029] Gottlieb, T. M. , and S. P. Jackson . 1993. “The DNA‐Dependent Protein Kinase: Requirement for DNA Ends and Association With Ku Antigen.” Cell 72: 131–142. 8422676 10.1016/0092-8674(93)90057-w

[gtc70076-bib-0030] Guturi, K. K. N. , M. Bohgaki , T. Bohgaki , et al. 2016. “RNF168 and USP10 Regulate Topoisomerase IIalpha Function via Opposing Effects on Its Ubiquitylation.” Nature Communications 7: 12638. 10.1038/ncomms12638 PMC5007378 27558965

[gtc70076-bib-0031] Haffner, M. C. , M. J. Aryee , A. Toubaji , et al. 2010. “Androgen‐Induced TOP2B‐Mediated Double‐Strand Breaks and Prostate Cancer Gene Rearrangements.” Nature Genetics 42: 668–675. 20601956 10.1038/ng.613 PMC3157086

[gtc70076-bib-0032] Heck, M. M. , W. N. Hittelman , and W. C. Earnshaw . 1988. “Differential Expression of DNA Topoisomerases I and II During the Eukaryotic Cell Cycle.” Proceedings of the National Academy of Sciences of the United States of America 85: 1086–1090. 2829215 10.1073/pnas.85.4.1086 PMC279709

[gtc70076-bib-0033] Herrero‐Ruiz, A. , P. M. Martinez‐Garcia , J. Terron‐Bautista , et al. 2021. “Topoisomerase IIalpha Represses Transcription by Enforcing Promoter‐Proximal Pausing.” Cell Reports 35: 108977. 33852840 10.1016/j.celrep.2021.108977 PMC8052185

[gtc70076-bib-0034] Hoa, N. N. , T. Shimizu , Z. W. Zhou , et al. 2016. “Mre11 Is Essential for the Removal of Lethal Topoisomerase 2 Covalent Cleavage Complexes.” Molecular Cell 64: 1010. 10.1016/j.molcel.2016.11.028 27912094

[gtc70076-bib-0035] Ishida, R. , M. Hamatake , R. A. Wasserman , J. L. Nitiss , J. C. Wang , and T. Andoh . 1995. “DNA Topoisomerase II Is the Molecular Target of Bisdioxopiperazine Derivatives ICRF‐159 and ICRF‐193 in *Saccharomyces cerevisiae* .” Cancer Research 55: 2299–2303. 7757979

[gtc70076-bib-0036] Jang, J. Y. , D. Kim , E. Im , and N. D. Kim . 2025. “Etoposide as a Key Therapeutic Agent in Lung Cancer: Mechanisms, Efficacy, and Emerging Strategies.” International Journal of Molecular Sciences 26: 796. 39859509 10.3390/ijms26020796 PMC11765581

[gtc70076-bib-0037] Jha, R. K. , D. Levens , and F. Kouzine . 2022. “Mechanical Determinants of Chromatin Topology and Gene Expression.” Nucleus 13: 94–115. 35220881 10.1080/19491034.2022.2038868 PMC8890386

[gtc70076-bib-0038] Jiang, N. , Y. Shen , X. Fei , et al. 2013. “Valosin‐Containing Protein Regulates the Proteasome‐Mediated Degradation of DNA‐PKcs in Glioma Cells.” Cell Death & Disease 4: e647. 23722536 10.1038/cddis.2013.171 PMC3674378

[gtc70076-bib-0039] Ju, B. G. , V. V. Lunyak , V. Perissi , et al. 2006. “A Topoisomerase IIbeta‐Mediated dsDNA Break Required for Regulated Transcription.” Science 312: 1798–1802. 16794079 10.1126/science.1127196

[gtc70076-bib-0040] Kilgas, S. , and K. Ramadan . 2023. “Inhibitors of the ATPase p97/VCP: From Basic Research to Clinical Applications.” Cell Chemical Biology 30: 3–21. 36640759 10.1016/j.chembiol.2022.12.007

[gtc70076-bib-0041] Kimura, K. , M. Saijo , M. Ui , and T. Enomoto . 1994. “Growth State‐ and Cell Cycle‐Dependent Fluctuation in the Expression of Two Forms of DNA Topoisomerase II and Possible Specific Modification of the Higher Molecular Weight Form in the M Phase.” Journal of Biological Chemistry 269: 1173–1176. 8288578

[gtc70076-bib-0042] King, I. F. , C. N. Yandava , A. M. Mabb , et al. 2013. “Topoisomerases Facilitate Transcription of Long Genes Linked to Autism.” Nature 501: 58–62. 23995680 10.1038/nature12504 PMC3767287

[gtc70076-bib-0043] Kumar‐Sinha, C. , S. A. Tomlins , and A. M. Chinnaiyan . 2008. “Recurrent Gene Fusions in Prostate Cancer.” Nature Reviews. Cancer 8: 497–511. 18563191 10.1038/nrc2402 PMC2711688

[gtc70076-bib-0044] Larsen, A. K. , A. E. Escargueil , and A. Skladanowski . 2003. “Catalytic Topoisomerase II Inhibitors in Cancer Therapy.” Pharmacology & Therapeutics 99: 167–181. 12888111 10.1016/s0163-7258(03)00058-5

[gtc70076-bib-0045] Larsen, N. B. , A. O. Gao , J. L. Sparks , et al. 2019. “Replication‐Coupled DNA‐Protein Crosslink Repair by SPRTN and the Proteasome in Xenopus Egg Extracts.” Molecular Cell 73: 574–588.e7. 30595436 10.1016/j.molcel.2018.11.024 PMC6375733

[gtc70076-bib-0046] Lee, K. C. , R. L. Bramley , I. G. Cowell , G. H. Jackson , and C. A. Austin . 2016. “Proteasomal Inhibition Potentiates Drugs Targeting DNA Topoisomerase II.” Biochemical Pharmacology 103: 29–39. 26794000 10.1016/j.bcp.2015.12.015 PMC5071433

[gtc70076-bib-0047] Lee, K. C. , K. Padget , H. Curtis , et al. 2012. “MRE11 Facilitates the Removal of Human Topoisomerase II Complexes From Genomic DNA.” Biology Open 1: 863–873. 23213480 10.1242/bio.20121834 PMC3507232

[gtc70076-bib-0048] Lee, K. C. , R. L. Swan , Z. Sondka , K. Padget , I. G. Cowell , and C. A. Austin . 2018. “Effect of TDP2 on the Level of TOP2‐DNA Complexes and SUMOylated TOP2‐DNA Complexes.” International Journal of Molecular Sciences 19: 2056. 30011940 10.3390/ijms19072056 PMC6073685

[gtc70076-bib-0049] Li, H. , Y. Wang , and X. Liu . 2008. “Plk1‐Dependent Phosphorylation Regulates Functions of DNA Topoisomerase IIalpha in Cell Cycle Progression.” Journal of Biological Chemistry 283: 6209–6221. 18171681 10.1074/jbc.M709007200

[gtc70076-bib-0050] Linders, A. N. , I. B. Dias , T. Lopez Fernandez , C. G. Tocchetti , N. Bomer , and P. Van der Meer . 2024. “A Review of the Pathophysiological Mechanisms of Doxorubicin‐Induced Cardiotoxicity and Aging.” NPJ Aging 10: 9. 38263284 10.1038/s41514-024-00135-7 PMC10806194

[gtc70076-bib-0051] Lopez‐Mosqueda, J. , K. Maddi , S. Prgomet , et al. 2016. “SPRTN Is a Mammalian DNA‐Binding Metalloprotease That Resolves DNA‐Protein Crosslinks.” eLife 5: e21491. 27852435 10.7554/eLife.21491 PMC5127644

[gtc70076-bib-0052] Lyu, Y. L. , J. E. Kerrigan , C. P. Lin , et al. 2007. “Topoisomerase IIbeta Mediated DNA Double‐Strand Breaks: Implications in Doxorubicin Cardiotoxicity and Prevention by Dexrazoxane.” Cancer Research 67: 8839–8846. 17875725 10.1158/0008-5472.CAN-07-1649

[gtc70076-bib-0053] Lyu, Y. L. , C. P. Lin , A. M. Azarova , L. Cai , J. C. Wang , and L. F. Liu . 2006. “Role of Topoisomerase IIbeta in the Expression of Developmentally Regulated Genes.” Molecular and Cellular Biology 26: 7929–7941. 16923961 10.1128/MCB.00617-06 PMC1636731

[gtc70076-bib-0054] Madabhushi, R. , F. Gao , A. R. Pfenning , et al. 2015. “Activity‐Induced DNA Breaks Govern the Expression of Neuronal Early‐Response Genes.” Cell 161: 1592–1605. 26052046 10.1016/j.cell.2015.05.032 PMC4886855

[gtc70076-bib-0055] Maede, Y. , H. Shimizu , T. Fukushima , et al. 2014. “Differential and Common DNA Repair Pathways for Topoisomerase I‐ and II‐Targeted Drugs in a Genetic DT40 Repair Cell Screen Panel.” Molecular Cancer Therapeutics 13: 214–220. 24130054 10.1158/1535-7163.MCT-13-0551 PMC3919527

[gtc70076-bib-0056] Manasanch, E. E. , and R. Z. Orlowski . 2017. “Proteasome Inhibitors in Cancer Therapy.” Nature Reviews. Clinical Oncology 14: 417–433. 10.1038/nrclinonc.2016.206 PMC5828026 28117417

[gtc70076-bib-0057] Mani, R. S. , S. A. Tomlins , K. Callahan , et al. 2009. “Induced Chromosomal Proximity and Gene Fusions in Prostate Cancer.” Science 326: 1230. 19933109 10.1126/science.1178124 PMC2935583

[gtc70076-bib-0058] Mao, Y. , S. D. Desai , C. Y. Ting , J. Hwang , and L. F. Liu . 2001. “26 S Proteasome‐Mediated Degradation of Topoisomerase II Cleavable Complexes.” Journal of Biological Chemistry 276: 40652–40658. 11546768 10.1074/jbc.M104009200

[gtc70076-bib-0059] Martensson, S. , J. Nygren , N. Osheroff , and O. Hammarsten . 2003. “Activation of the DNA‐Dependent Protein Kinase by Drug‐Induced and Radiation‐Induced DNA Strand Breaks.” Radiation Research 160: 291–301. 12926987 10.1667/0033-7587(2003)160[0291:aotdpk]2.0.co;2

[gtc70076-bib-0060] McClendon, A. K. , A. C. Rodriguez , and N. Osheroff . 2005. “Human Topoisomerase IIalpha Rapidly Relaxes Positively Supercoiled DNA: Implications for Enzyme Action Ahead of Replication Forks.” Journal of Biological Chemistry 280: 39337–39345. 16188892 10.1074/jbc.M503320200

[gtc70076-bib-0061] Meaburn, K. J. , T. Misteli , and E. Soutoglou . 2007. “Spatial Genome Organization in the Formation of Chromosomal Translocations.” Seminars in Cancer Biology 17: 80–90. 17137790 10.1016/j.semcancer.2006.10.008 PMC1805052

[gtc70076-bib-0062] Meyer, H. , M. Bug , and S. Bremer . 2012. “Emerging Functions of the VCP/p97 AAA‐ATPase in the Ubiquitin System.” Nature Cell Biology 14: 117–123. 22298039 10.1038/ncb2407

[gtc70076-bib-0063] Mistry, A. R. , C. A. Felix , R. J. Whitmarsh , et al. 2005. “DNA Topoisomerase II in Therapy‐Related Acute Promyelocytic Leukemia.” New England Journal of Medicine 352: 1529–1538. 15829534 10.1056/NEJMoa042715

[gtc70076-bib-0064] Mondal, N. , and J. D. Parvin . 2001. “DNA Topoisomerase IIalpha Is Required for RNA Polymerase II Transcription on Chromatin Templates.” Nature 413: 435–438. 11574892 10.1038/35096590

[gtc70076-bib-0065] Morocz, M. , E. Zsigmond , R. Toth , M. Z. Enyedi , L. Pinter , and L. Haracska . 2017. “DNA‐Dependent Protease Activity of Human Spartan Facilitates Replication of DNA‐Protein Crosslink‐Containing DNA.” Nucleic Acids Research 45: 3172–3188. 28053116 10.1093/nar/gkw1315 PMC5389635

[gtc70076-bib-0066] Nakamura, K. , T. Kogame , H. Oshiumi , et al. 2010. “Collaborative Action of Brca1 and CtIP in Elimination of Covalent Modifications From Double‐Strand Breaks to Facilitate Subsequent Break Repair.” PLoS Genetics 6: e1000828. 20107609 10.1371/journal.pgen.1000828 PMC2809774

[gtc70076-bib-0067] Nicolette, M. L. , K. Lee , Z. Guo , et al. 2010. “Mre11‐Rad50‐Xrs2 and Sae2 Promote 5′ Strand Resection of DNA Double‐Strand Breaks.” Nature Structural & Molecular Biology 17: 1478–1485. 10.1038/nsmb.1957 PMC3059534 21102445

[gtc70076-bib-0068] Nielsen, C. F. , T. Zhang , M. Barisic , P. Kalitsis , and D. F. Hudson . 2020. “Topoisomerase IIalpha Is Essential for Maintenance of Mitotic Chromosome Structure.” Proceedings of the National Academy of Sciences of the United States of America 117: 12131–12142. 32414923 10.1073/pnas.2001760117 PMC7275761

[gtc70076-bib-0069] Nitiss, J. L. 2009a. “DNA Topoisomerase II and Its Growing Repertoire of Biological Functions.” Nature Reviews. Cancer 9: 327–337. 19377505 10.1038/nrc2608 PMC2730144

[gtc70076-bib-0070] Nitiss, J. L. 2009b. “Targeting DNA Topoisomerase II in Cancer Chemotherapy.” Nature Reviews. Cancer 9: 338–350. 19377506 10.1038/nrc2607 PMC2748742

[gtc70076-bib-0071] Olmedo‐Pelayo, J. , D. Rubio‐Contreras , and F. Gomez‐Herreros . 2020. “Canonical Non‐Homologous End‐Joining Promotes Genome Mutagenesis and Translocations Induced by Transcription‐Associated DNA Topoisomerase 2 Activity.” Nucleic Acids Research 48: 9147–9160. 32749454 10.1093/nar/gkaa640 PMC7498328

[gtc70076-bib-0072] Paull, T. T. 2018. “20 Years of Mre11 Biology: No End in Sight.” Molecular Cell 71: 419–427. 30057197 10.1016/j.molcel.2018.06.033

[gtc70076-bib-0073] Paull, T. T. , E. P. Rogakou , V. Yamazaki , C. U. Kirchgessner , M. Gellert , and W. M. Bonner . 2000. “A Critical Role for Histone H2AX in Recruitment of Repair Factors to Nuclear Foci After DNA Damage.” Current Biology 10: 886–895. 10959836 10.1016/s0960-9822(00)00610-2

[gtc70076-bib-0074] Pendleton, M. , R. H. Lindsey Jr. , C. A. Felix , D. Grimwade , and N. Osheroff . 2014. “Topoisomerase II and Leukemia.” Annals of the New York Academy of Sciences 1310: 98–110. 24495080 10.1111/nyas.12358 PMC3961513

[gtc70076-bib-0075] Pommier, Y. , E. Leo , H. Zhang , and C. Marchand . 2010. “DNA Topoisomerases and Their Poisoning by Anticancer and Antibacterial Drugs.” Chemistry & Biology 17: 421–433. 20534341 10.1016/j.chembiol.2010.04.012 PMC7316379

[gtc70076-bib-0076] Pommier, Y. , A. Nussenzweig , S. Takeda , and C. Austin . 2022. “Human Topoisomerases and Their Roles in Genome Stability and Organization.” Nature Reviews. Molecular Cell Biology 23: 407–427. 35228717 10.1038/s41580-022-00452-3 PMC8883456

[gtc70076-bib-0077] Qi, S. , Z. Shi , and H. Yu . 2025. “Genome Folding by Cohesin.” Current Opinion in Genetics & Development 91: 102310. 39827577 10.1016/j.gde.2025.102310

[gtc70076-bib-0078] Quennet, V. , A. Beucher , O. Barton , S. Takeda , and M. Lobrich . 2011. “CtIP and MRN Promote Non‐Homologous End‐Joining of Etoposide‐Induced DNA Double‐Strand Breaks in G1.” Nucleic Acids Research 39: 2144–2152. 21087997 10.1093/nar/gkq1175 PMC3064790

[gtc70076-bib-0079] Robison, J. G. , K. Dixon , and J. J. Bissler . 2007. “Cell Cycle‐and Proteasome‐Dependent Formation of Etoposide‐Induced Replication Protein A (RPA) or Mre11/Rad50/Nbs1 (MRN) Complex Repair Foci.” Cell Cycle 6: 2399–2407. 17700069 10.4161/cc.6.19.4772

[gtc70076-bib-0080] Rogakou, E. P. , D. R. Pilch , A. H. Orr , V. S. Ivanova , and W. M. Bonner . 1998. “DNA Double‐Stranded Breaks Induce Histone H2AX Phosphorylation on Serine 139.” Journal of Biological Chemistry 273: 5858–5868. 9488723 10.1074/jbc.273.10.5858

[gtc70076-bib-0081] Ryan, D. P. , B. H. O'Neil , J. G. Supko , et al. 2006. “A Phase I Study of Bortezomib Plus Irinotecan in Patients With Advanced Solid Tumors.” Cancer 107: 2688–2697. 17075878 10.1002/cncr.22280

[gtc70076-bib-0082] Saha, L. K. , S. Saha , X. Yang , et al. 2023. “Replication‐Associated Formation and Repair of Human Topoisomerase IIIalpha Cleavage Complexes.” Nature Communications 14: 1925. 10.1038/s41467-023-37498-6 PMC10079683 37024461

[gtc70076-bib-0083] Sakasai, R. , Y. Sunatani , T. Matsui , and K. Iwabuchi . 2025. “Valosin‐Containing Protein Mediates DNA‐Dependent Protein Kinase Activation in Response to DNA Topoisomerase II‐Associated DNA Double‐Strand Breaks.” Journal of Biochemistry 178: 51–60. 40366651 10.1093/jb/mvaf025

[gtc70076-bib-0084] Sakasai, R. , H. Teraoka , and R. S. Tibbetts . 2010. “Proteasome Inhibition Suppresses DNA‐Dependent Protein Kinase Activation Caused by Camptothecin.” DNA Repair (Amst) 9: 76–82. 19959400 10.1016/j.dnarep.2009.10.008 PMC2818427

[gtc70076-bib-0085] Sartori, A. A. , C. Lukas , J. Coates , et al. 2007. “Human CtIP Promotes DNA End Resection.” Nature 450: 509–514. 17965729 10.1038/nature06337 PMC2409435

[gtc70076-bib-0086] Sasanuma, H. , M. Tsuda , S. Morimoto , et al. 2018. “BRCA1 Ensures Genome Integrity by Eliminating Estrogen‐Induced Pathological Topoisomerase II‐DNA Complexes.” Proceedings of the National Academy of Sciences of the United States of America 115: E10642–E10651. 30352856 10.1073/pnas.1803177115 PMC6233096

[gtc70076-bib-0087] Schellenberg, M. J. , J. A. Lieberman , A. Herrero‐Ruiz , et al. 2017. “ZATT (ZNF451)‐mediated Resolution of Topoisomerase 2 DNA‐Protein Cross‐Links.” Science 357: 1412–1416. 28912134 10.1126/science.aam6468 PMC5623066

[gtc70076-bib-0088] Schellenberg, M. J. , L. Perera , C. N. Strom , et al. 2016. “Reversal of DNA Damage Induced Topoisomerase 2 DNA‐Protein Crosslinks by Tdp2.” Nucleic Acids Research 44: 3829–3844. 27060144 10.1093/nar/gkw228 PMC4857006

[gtc70076-bib-0089] Sciascia, N. , W. Wu , D. Zong , et al. 2020. “Suppressing Proteasome Mediated Processing of Topoisomerase II DNA‐Protein Complexes Preserves Genome Integrity.” eLife 9: e53447. 32057297 10.7554/eLife.53447 PMC7089766

[gtc70076-bib-0090] Shu, J. , D. Cui , Y. Ma , X. Xiong , Y. Sun , and Y. Zhao . 2020. “SCF(Beta‐TrCP)‐mediated Degradation of TOP2beta Promotes Cancer Cell Survival in Response to Chemotherapeutic Drugs Targeting Topoisomerase II.” Oncogene 9: 8. 10.1038/s41389-020-0196-1 PMC6997367 32015321

[gtc70076-bib-0091] Sin, C. F. , and P. M. Man . 2021. “The Role of Proteasome Inhibitors in Treating Acute Lymphoblastic Leukaemia.” Frontiers in Oncology 11: 802832. 35004327 10.3389/fonc.2021.802832 PMC8733464

[gtc70076-bib-0092] Stingele, J. , R. Bellelli , F. Alte , et al. 2016. “Mechanism and Regulation of DNA‐Protein Crosslink Repair by the DNA‐Dependent Metalloprotease SPRTN.” Molecular Cell 64: 688–703. 27871365 10.1016/j.molcel.2016.09.031 PMC5128726

[gtc70076-bib-0093] Sun, Y. , L. M. Miller Jenkins , Y. P. Su , K. C. Nitiss , J. L. Nitiss , and Y. Pommier . 2020. “A Conserved SUMO Pathway Repairs Topoisomerase DNA‐Protein Cross‐Links by Engaging Ubiquitin‐Mediated Proteasomal Degradation.” Science Advances 6: eaba6290. 33188014 10.1126/sciadv.aba6290 PMC7673754

[gtc70076-bib-0094] Sun, Y. , E. Soans , M. Mishina , et al. 2022. “Requirements for MRN Endonuclease Processing of Topoisomerase II‐Mediated DNA Damage in Mammalian Cells.” Frontiers in Molecular Biosciences 9: 1007064. 36213114 10.3389/fmolb.2022.1007064 PMC9537633

[gtc70076-bib-0095] Swan, R. L. , I. G. Cowell , and C. A. Austin . 2021. “A Role for VCP/p97 in the Processing of Drug‐Stabilized TOP2‐DNA Covalent Complexes.” Molecular Pharmacology 100: 57–62. 33941661 10.1124/molpharm.121.000262 PMC7611185

[gtc70076-bib-0096] Swan, R. L. , L. L. K. Poh , I. G. Cowell , and C. A. Austin . 2020. “Small Molecule Inhibitors Confirm Ubiquitin‐Dependent Removal of TOP2‐DNA Covalent Complexes.” Molecular Pharmacology 98: 222–233. 32587095 10.1124/mol.119.118893 PMC7416847

[gtc70076-bib-0097] Szponar, J. , E. Ciechanski , M. Ciechanska , J. Dudka , and S. Mandziuk . 2024. “Evolution of Theories on Doxorubicin‐Induced Late Cardiotoxicity‐Role of Topoisomerase.” International Journal of Molecular Sciences 25: 13567. 39769331 10.3390/ijms252413567 PMC11678604

[gtc70076-bib-0098] Tammaro, M. , P. Barr , B. Ricci , and H. Yan . 2013. “Replication‐Dependent and Transcription‐Dependent Mechanisms of DNA Double‐Strand Break Induction by the Topoisomerase 2‐Targeting Drug Etoposide.” PLoS One 8: e79202. 24244448 10.1371/journal.pone.0079202 PMC3820710

[gtc70076-bib-0099] Tang, W. , G. Su , J. Li , et al. 2014. “Enhanced Anti‐Colorectal Cancer Effects of Carfilzomib Combined With CPT‐11 via Downregulation of Nuclear Factor‐kappaB in Vitro and in Vivo.” International Journal of Oncology 45: 995–1010. 24968890 10.3892/ijo.2014.2513 PMC4121410

[gtc70076-bib-0100] Thakurela, S. , A. Garding , J. Jung , D. Schubeler , L. Burger , and V. K. Tiwari . 2013. “Gene Regulation and Priming by Topoisomerase IIalpha in Embryonic Stem Cells.” Nature Communications 4: 2478. 10.1038/ncomms3478 24072229

[gtc70076-bib-0101] Uuskula‐Reimand, L. , H. Hou , P. Samavarchi‐Tehrani , et al. 2016. “Topoisomerase II Beta Interacts With Cohesin and CTCF at Topological Domain Borders.” Genome Biology 17: 182. 27582050 10.1186/s13059-016-1043-8 PMC5006368

[gtc70076-bib-0102] Uuskula‐Reimand, L. , and M. D. Wilson . 2022. “Untangling the Roles of TOP2A and TOP2B in Transcription and Cancer.” Science Advances 8: eadd4920. 36322662 10.1126/sciadv.add4920 PMC9629710

[gtc70076-bib-0103] van den Boom, J. , M. Wolf , L. Weimann , et al. 2016. “VCP/p97 Extracts Sterically Trapped Ku70/80 Rings From DNA in Double‐Strand Break Repair.” Molecular Cell 64: 189–198. 27716483 10.1016/j.molcel.2016.08.037 PMC5161236

[gtc70076-bib-0104] Vann, K. R. , A. A. Oviatt , and N. Osheroff . 2021. “Topoisomerase II Poisons: Converting Essential Enzymes Into Molecular Scissors.” Biochemistry 60: 1630–1641. 34008964 10.1021/acs.biochem.1c00240 PMC8209676

[gtc70076-bib-0105] Vaz, B. , M. Popovic , J. A. Newman , et al. 2016. “Metalloprotease SPRTN/DVC1 Orchestrates Replication‐Coupled DNA‐Protein Crosslink Repair.” Molecular Cell 64: 704–719. 27871366 10.1016/j.molcel.2016.09.032 PMC5128727

[gtc70076-bib-0106] Venkatachalam, A. , and S. H. Kaufmann . 2025. “Targeting DNA Topoisomerase I for the Treatment of Cancer: Past, Present and Future.” Journal of Molecular Biology: 169401. 10.1016/j.jmb.2025.169401. 40848933 PMC12449767

[gtc70076-bib-0107] von Metzler, I. , U. Heider , M. Mieth , et al. 2009. “Synergistic Interaction of Proteasome and Topoisomerase II Inhibition in Multiple Myeloma.” Experimental Cell Research 315: 2471–2478. 19410573 10.1016/j.yexcr.2009.04.019

[gtc70076-bib-0108] Wang, W. , S. Saha , X. Yang , Y. Pommier , and S. N. Huang . 2023. “Identification and Characterization of Topoisomerase III Beta Poisons.” Proceedings of the National Academy of Sciences of the United States of America 120: e2218483120. 37579177 10.1073/pnas.2218483120 PMC10450851

[gtc70076-bib-0109] Wei, Y. , L. X. Diao , S. Lu , et al. 2017. “SUMO‐Targeted DNA Translocase Rrp2 Protects the Genome From Top2‐Induced DNA Damage.” Molecular Cell 66: 581–596.e6. 28552615 10.1016/j.molcel.2017.04.017

[gtc70076-bib-0110] Weickert, P. , H. Y. Li , M. J. Gotz , et al. 2023. “SPRTN Patient Variants Cause Global‐Genome DNA‐Protein Crosslink Repair Defects.” Nature Communications 14: 352. 10.1038/s41467-023-35988-1 PMC9867749 36681662

[gtc70076-bib-0111] Willmore, E. , S. de Caux , N. J. Sunter , et al. 2004. “A Novel DNA‐Dependent Protein Kinase Inhibitor, NU7026, Potentiates the Cytotoxicity of Topoisomerase II Poisons Used in the Treatment of Leukemia.” Blood 103: 4659–4665. 15010369 10.1182/blood-2003-07-2527

[gtc70076-bib-0112] Winick, N. J. , R. W. McKenna , J. J. Shuster , et al. 1993. “Secondary Acute Myeloid Leukemia in Children With Acute Lymphoblastic Leukemia Treated With Etoposide.” Journal of Clinical Oncology 11: 209–217. 8426196 10.1200/JCO.1993.11.2.209

[gtc70076-bib-0113] Xiao, H. , Y. Mao , S. D. Desai , et al. 2003. “The Topoisomerase IIbeta Circular Clamp Arrests Transcription and Signals a 26S Proteasome Pathway.” Proceedings of the National Academy of Sciences of the United States of America 100: 3239–3244. 12629207 10.1073/pnas.0736401100 PMC152276

[gtc70076-bib-0114] Yao, Q. , L. Zhu , Z. Shi , S. Banerjee , and C. Chen . 2025. “Topoisomerase‐Modulated Genome‐Wide DNA Supercoiling Domains Colocalize With Nuclear Compartments and Regulate Human Gene Expression.” Nature Structural & Molecular Biology 32: 48–61. 10.1038/s41594-024-01377-5 39152238

[gtc70076-bib-0115] Zeng, Z. , F. Cortes‐Ledesma , S. F. El Khamisy , and K. W. Caldecott . 2011. “TDP2/TTRAP Is the Major 5′‐Tyrosyl DNA Phosphodiesterase Activity in Vertebrate Cells and Is Critical for Cellular Resistance to Topoisomerase II‐Induced DNA Damage.” Journal of Biological Chemistry 286: 403–409. 21030584 10.1074/jbc.M110.181016 PMC3012998

[gtc70076-bib-0116] Zhang, A. , Y. L. Lyu , C. P. Lin , et al. 2006. “A Protease Pathway for the Repair of Topoisomerase II‐DNA Covalent Complexes.” Journal of Biological Chemistry 281: 35997–36003. 16973621 10.1074/jbc.M604149200

[gtc70076-bib-0117] Zhang, H. , Y. Xiong , Y. Sun , et al. 2023. “RAD54L2‐Mediated DNA Damage Avoidance Pathway Specifically Preserves Genome Integrity in Response to Topoisomerase 2 Poisons.” Science Advances 9: eadi6681. 38055811 10.1126/sciadv.adi6681 PMC10699775

[gtc70076-bib-0118] Zhang, S. , X. Liu , T. Bawa‐Khalfe , et al. 2012. “Identification of the Molecular Basis of Doxorubicin‐Induced Cardiotoxicity.” Nature Medicine 18: 1639–1642. 10.1038/nm.2919 23104132

